# Comparison of COVID-19 vaccine policies and their effectiveness in Korea, Japan, and Singapore

**DOI:** 10.1186/s12939-023-02034-x

**Published:** 2023-10-20

**Authors:** Mengyuan Ma, Leiyu Shi, Meiheng Liu, Junyan Yang, Wanzhen Xie, Gang Sun

**Affiliations:** 1 Department of Health Management, School of Health Management, Southern Medical University, Guangzhou, 510515 China; 2 Department of Health Policy and Management, Bloomberg School of Public Health, Johns Hopkins University, Baltimore, MD 21205 USA

**Keywords:** COVID-19, Vaccine policies, Vaccination, Effective reproduction rate

## Abstract

**Background:**

This study aimed to analyze coronavirus disease 2019 (COVID-19)vaccine policies and effectiveness in Korea, Japan, and Singapore, thereby providing empirical experience for vaccination and response to similar public health emergencies.

**Methods:**

The study systematically summarized the COVID-19 vaccine policies in Korea, Japan, and Singapore through public information from the Our World in Data website and the official websites of the Ministries of Health in these three countries.Total vaccinations, COVID-19 vaccination rates, rates of fully vaccinated, rates of boostervaccinated, and total confifirmed cases were selected for cross-sectional comparison of COVID-19 vaccination in these three countries. Combining the basic characteristics of these three countries, daily cases per million, daily deaths per million, and the effective reproduction rate were calculated to measure the effectiveness of COVID-19 vaccine policies implementation in each of these three countries

**Results:**

The countermeasures against the COVID-19 in Korea, Japan, and Singapore, although seemingly different on the surface, have all taken an aggressive approach. There are large similarities in the timing of the start of COVID-19 vaccination, the type of vaccine, how vaccine appointments are made, and whether vaccination are free, and all had high vaccination rates. A systematic comparison of the anti-epidemic practices in the three East Asian countries revealed that all three countries experienced more than one outbreak spike due to the spread of new mutant strains after the start of mass vaccination with COVID-19 vaccination, but that vaccination played a positive role in reducing the number of deaths and stabilizing the effective reproduction rate.

**Conclusions:**

This study comparatively analyzed the COVID-19 vaccine policies and their effects in South Korea, Japan, and Singapore, and found that there is a common set of logical combinations behind the seemingly different strategies of these three countries. Therefore, in the process of combating COVID-19, countries can learn from the successful experience of combating the epidemic and continue to strengthen the implementation of vaccination programs, as well as adjusting public perceptions to reduce the level of vaccine hesitancy, enhance the motivation for vaccination, and improve the coverage of COVID-19 vaccine based on different cultural factors, which remains the direction for future development.

## Introduction

The worldwide pandemic of coronavirus disease 2019 (COVID-19) is the most serious public health threat since the 1918 H1N1 influenza pandemic [1]. The first case of COVID-19 caused by SARS-CoV-2 was diagnosed in Wuhan, China, and the infection spread rapidly to other parts of China and then to the world within a short period of time [2]; On January 30, 2020, the World Health Organization (WHO) declared the COVID—19 constitutes a "Public Health Emergencies of International Concern", the highest level of warning that WHO can issue under the International Health Regulations [3]; On March 11, 2020,WHO declared it constitutes a global pandemic and called on governments to take active and urgent preventive and control measures, balancing health protection with reduced disruption and respect for human rights [4]. Until May 5, 2023, when the WHO declares that the COVID—19 no longer constitutes a "public health emergency of international concern" and lifts the highest level of alert issued on January 30, 2020, marking a "major turning point" in the global treatment of the crisis accordingly [5]; At the 76th World Health Assembly of the WHO in Geneva, Switzerland, May 21, 2023, local time, WHO Director-General Tan Desai stressed that although it no longer constitutes a public health emergency, the virus continues to mutate and the next global pandemic should be guarded against. Over the past three years, the world had experienced several rounds of epidemic peaks caused by mutated strains of Alpha, Delta, and Omicron, with WHO data showing that more than 760 million confirmed cases and more than 6.9 million deaths have been reported worldwide. At the same time, the global pandemic has posed significant threats and challenges to global economic development, health care systems, and public emergency management.

Since the COVID-19 pandemic, despite the various measures taken by countries to control the epidemic, the high transmission capacity of the virus, the rapid mutation rate, the general susceptibility of the population and the many asymptomatic infected persons have put enormous pressure on global public health, vaccination has become the most cost-effective means of controlling outbreaks [6]. The development of a vaccine for the SARS-CoV-2 virus in less than a year was an unprecedented scientific feat, the WHO released the Global COVID-19 Immunization Strategy, which states that COVID-19 vaccination has dramatically changed the course of pandemics and saved the lives of tens of millions of people worldwide [7, 8]. Starting with the first Pfizer/BioNTech vaccine to pass the WHO emergency access and expand its supply for worldwide distribution on December 31, 2020, and with the dramatically shortened vaccine development cycle in the context of big medical data as a catalyst, the process of developing vaccines is moving at an unprecedented pace. Currently, there are at least 18 different types of COVID-19 vaccines that have received emergency approval from at least one government pharmaceutical regulatory agency worldwide. As of June 2023, 13.41 billion doses of vaccine have been administered globally, with more than 70% of the population having received at least one dose of COVID-19 vaccine, 64.38% of the population having completed the basic vaccination, and 34.68% of the population having completed at least one booster dose of the vaccine.

Although each country fights the epidemic differently, the external shocks and expectations to end the epidemic are the same for all countries. Therefore, we need to understand why the same effects occur in different countries, that is, what is the common logic behind the seemingly different strategies. The central question of this study is: Why did the three countries achieve similar results with different policy strategies? Thus, this article is a study in search of commonalities. Finding and refining the basic principles for fighting the epidemic can provide important guidance for future epidemic prevention and control [9]. Considering the progress in vaccination, the theoretical basis of whether the strategies and measures in vaccine procurement are all through diversified procurement, how the partnership with manufacturers is, and the differences in vaccination policies, as well as the empirical basis of the similarity in the timing of the epidemic waves, the fact that the vaccination rates are all among the leading rates globally, and the fact that the basic conditions of the countries are similar, and that the economic recoveries are all progressing in a positive direction. Taking the vaccination rate as the main basis, this study selected three Asian countries, Korea, Japan and Singapore, and started from the vaccination policies of Korea, Japan and Singapore, and categorized and summarized the means of vaccination in these countries, and systematically made direct comparisons between the conditions of each country, analyzing and comparing the basic conditions and effects of vaccination in these three countries, and judging whether each country has adopted the same practices in these commonalities, and then reveal the key logic behind these practices, with a view to providing guidance for other countries to carry out COVID-19 vaccination work, advancing the progress of global vaccination, and providing empirical experience for responding to similar public health emergencies.

## Materials and methods

### Data collection

The COVID-19 epidemiological data used in this study were obtained from publicly available information on the Our World in Data website and the official websites of the national health ministries of Korea, Japan, and Singapore. The data were selected for the period from the start of COVID-19 vaccination in each of the three countries to May 5, 2023. Total vaccinations, COVID-19 vaccination rates, rates of fully vaccinated, rates of booster-vaccinated, and total confifirmed cases were selected for cross-sectional comparison of COVID-19 vaccination in these three countries. Daily cases per million, daily deaths per million, and the effective reproduction rate (Rt) were calculated to measure the effectiveness of COVID-19 vaccine policies implementation in each of these three countries.

Total vaccinations are the sum of the basic and booster doses of the COVID-19 vaccine and are the total number of doses of COVID-19 vaccine administered. COVID-19 vaccination rates are the proportion of the population that has received at least one dose of COVID-19 vaccine, including the proportion of the population that is fully vaccinated and the proportion of the population that is partially vaccinated. Daily cases per million is the number of new confifirmed cases per million population per day for each country. Daily deaths per million is the number of new deaths per million population per day for each country. The effective reproduction rate is an indicator of how many people are infected by one individual. If Rt = 2, this implies that each infected person transmits the virus to two others, resulting in an uncontrolled epidemic [10]. Rt can assess the current dynamics of infectious disease transmission in a timely manner, reflflecting the severity of the pandemic, may be inflfluenced by policy and population immunity levels. Achieving a value of Rt < 1 is a necessary condition to stop the spread of virus [11].

### Policies information

The COVID-19 vaccine policies of Korea, Japan and Singapore are obtained from official WHO documents, survey reports, and official government websites and official documents of the three countries, such as Ministry of Health of Singapore, Japanese ministry of health, labor and welfare, Korean Ministry of Health and Welfare, etc. The COVID-19 vaccine policy extracted from this study includes the following aspects: basic vaccination program, vaccine procurement and supply, vaccine development, vaccine booster vaccination, vaccination of underage groups, mandatory vaccination, incentive vaccination, and vaccination support policies.

Finally, we conducted a cross-sectional comparison of basic COVID-19 vaccination in Korea, Japan, and Singapore, summarized the COVID-19 vaccination policies in each of these three countries, plotted the epidemiological curves of each of these three countries, and labeled the major vaccination policies to assess the effectiveness of the COVID-19 vaccination policies adopted by each country and to examine their commonalities.

## Results

### Basic information on COVID-19 vaccination in the three countries

Cross-country comparative studies need to be based on an understanding of the underlying country context, otherwise they tend to yield unrealistic policy recommendations [12]. First, in terms of the epidemic cycle, the three countries were similar at the beginning of vaccination, were in a similar epidemic cycle, and all adopted an aggressive response strategy. Second, in terms of population size, the three countries have different population sizes. As shown in Table 1, Korea has a total population of 51.27 million, which is equivalent to five times the size of Japan's population and approximates to 10 times the size of Singapore's total population, while its population is equivalent to the second largest city in Korea, thus making Singapore's population density more than ten times the population density of Korea and Japan. Again, in terms of demographics, the average life expectancy of all three countries is highly the same at around 84 years old, with Japan having the highest level of aging with nearly 27% of the population over 65 years old, and Singapore and Korea having similar levels of aging with 13% of the population over 65 years old. Finally, in terms of economic levels and regional disparities, Singapore, by comparison, has the highest per capita gross national product (GDP), which is more than twenty times the per capita GDP of Korea and Japan. Based on these national circumstances, this means that their strategic choices will face different constraints, but unexpectedly, the COVID-19 vaccination policies of the three countries are slightly different, but the overall pace is the same. As can be seen by Table 1, the basic COVID-19 vaccination profiles of Korea, Japan, and Singapore1 are very similar. In terms of starting time, Singapore was the first to start mass vaccination, followed by Japan and Korea two months later. In addition, all three countries received the COVID-19 vaccine from Pfizer/BioNTech, Moderna, AstraZeneca, and Novavax. The COVID-19 vaccination policies in all three countries are relatively strict, and all have phased in vaccine appointments and full free vaccination. In the early stages, vaccine appointments were made by age group and only for citizens within a specified age range, and in the middle stages, as vaccination rates increased and vaccine supplies gradually became sufficient, the age requirement for vaccine appointments was removed and universal free vaccination was implemented.

**Table 1:** Basic information on COVID-19 vaccination in Korea, Japan and Singapore

	Korea	Japan	Singapore
Population (millions)	51.27	12.65	5.85
Density of population(P/km^2^)	359	390	8554
GDP per capita ($ million)	3.22	4.13	8.27
Percentage of population over 65 years old(%)	13.91	27.05	12.92
Expected life expectancy (years)	83.03	84.63	83.62
Cumulative number of confirmed cases (/ million)	603,348.77	272,420.29	424,296.73
Cumulative number of deaths (/ million)	666.05	601.96	305.48
Start date of vaccination	February 26, 2021	February 17, 2021	December 30, 2020
Cumulative vaccinations (millions)	129.65	385.75	14.72
COVID-19 vaccination rate(%)	86.43	84.47	91.55
Share of people with fully vaccinated(%)	85.64	83.40	90.85
Share of people only partly vaccinated(%)	0.79	1.07	0.70
Share of people with booster vaccinated(%)	79.76	141.71	78.77
Vaccines Appointment	Vaccine appointments are made online or by phone	Vaccine appointments are made online or by phone	Vaccine appointments are made online or by phone
Free vaccination or not	Free vaccination for the entire population	Free vaccination for the entire population	Free vaccination for the entire population
Vaccines administered	Pfizer/BioNTech, Moderna, AstraZeneca, Novavax, Johnson&Johnson	Pfizer/BioNTech, Moderna, AstraZeneca	Pfizer-BioNTech/Comirnaty Moderna/Spikevax Novavax/Nuvaxovid Sinovac-CoronaVac

The start date of vaccination is the local time of each country

**Fig. 1:**
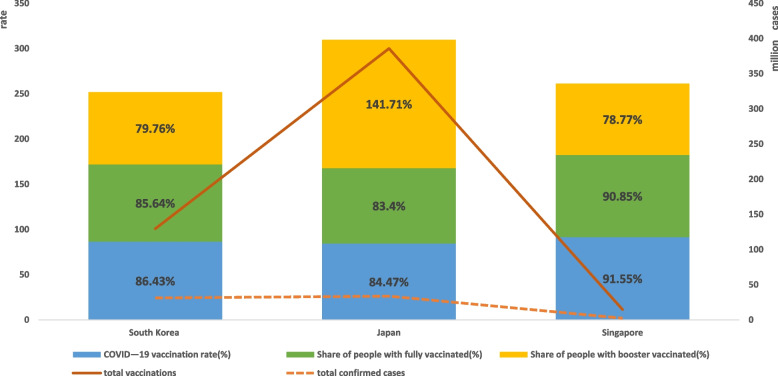
Total confirmed cases and COVID-19 vaccination rates in the three countries. Note1: COVID-19 vaccination rate, Share of people with fully/booster vaccinated refer to main axis(left). Total confirmed cases and total vaccinations refer to secondary axis(right). Note2: Given that Share of people only partly vaccinated values are too far away from the rest, their data are not represented on the bar chart

As seen by Fig. 1, Singapore has the highest level of COVID-19 vaccination among the three countries. Although the number of vaccine doses is lower than Korea and Japan due to the upper limit of population size, the overall vaccination rate, the proportion of population fully vaccinated, and the proportion of population with vaccine booster shots are the highest. Japan has the highest total number of vaccination doses among the three countries, but both the vaccination rate and the percentage of the population fully vaccinated are lower than those of Singapore and Korea due to prior vaccine supply and domestic willingness to vaccinate, while the percentage of the population receiving vaccine booster shots in Japan is higher than that of Singapore and Korea. Overall, Korea, Japan, and Singapore, three of the more developed Asian countries, maintain approximate vaccination levels at high levels. There was a large difference in the cumulative number of confirmed cases between the three countries, with Japan having the fewest and Korea having the most, more than twice as many as Japan and more than 1.5 times as many as Singapore. Korea and Japan had similar cumulative deaths per million, nearly twice the number of cumulative deaths per million in Singapore.

### Core COVID-19 vaccine policies of the three countries

#### Korea

Korea launched a large-scale COVID-19 vaccination on February 26, 2021, as can be seen by Table 2, dividing the target population into six groups considering susceptibility risk and severe disease factors, and determining the type of vaccine, timing and method of vaccination for each target population, taking into account the target population, vaccine characteristics, introduction time and quantity, starting with the vaccination of medical staff with AstraZeneca vaccine, and expanding sequentially from the elderly group and socially necessary manpower to the population over 18 years old and the underage group. As of December 21, 2021, Korea has a world-leading rate of at least one vaccination of 84.95%, a complete vaccination rate of 82.05% for basic vaccines, and a vaccination rate of about 80% for all geographic areas within Korea. However, since most of the COVID-19 vaccine in Korea relies on imports from Europe and the United States, and side effects caused by vaccination are common in Korea, the uncontrollable nature of the vaccine has caused the Korean government to lose a lot of credibility. This was not effectively mitigated until June 29, 2022, when Korea's first COVID-19 vaccine, developed by SK Bioscience, was licensed by the Ministry of Food and Drug Safety's SkyCorvione vaccine program. At the same time, the Korean government has been tracking and publicly announcing adverse vaccination events since vaccination to compensate for the loss of credibility, and has introduced and continuously improved compensation for vaccine damages. Since November 1, 2021, the continuous mutation of the COVID-19 strain, coupled with the declining effectiveness of the vaccine over time, has led to a rapid increase in the number of confirmed COVID-19 cases, the highest since the COVID-19 outbreak, and a significant increase in the number of deaths, showing a rapid increase. In order to interrupt the delta mutation epidemic in time to prepare for the Omicron mutation epidemic, the Korean government has implemented a mandatory vaccination program for those who meet the vaccine interval. In February 2022, based on the analysis of data on cumulative risk ratios in the domestic cohort, and based on the objective fact that the third vaccination with the omicron mutation was 70–80% effective within 3 months as found in the UK vaccination efficacy analysis, the Korean government adopted a population-specific additional vaccine program for high-risk groups (immunocompromised individuals).

**Table 2:** COVID-19 vaccine policies of Korea

Aspects	COVID-19 vaccine policies
Basic vaccination plan	**1. Phase I vaccination:** February 26, 2021 for high-risk medical institution employees, nursing home elderly and staff **2. Phase II vaccination:** from March 15, 2021 for the elderly over 65 years old, disabled welfare facility employees, wider medical and nursing staff, chronic disease patients (high-risk groups of dialysis patients, chronic kidney disease, etc.), and socially necessary manpower (police, marine police, firefighters, soldiers, aircrews, etc.) **3. Phase III vaccination:** General public aged 18–64 from July 19, 2021 **4. Phase IV vaccination:** Children and adolescents aged 12–17 years and pregnant women from August 30, 2021 **5. Phase V Vaccination:** All unvaccinated people over 18 years of age from October 1, 2021
Vaccine procurement and supply	1. February 2021, the Korea Center for Disease Control and Prevention (KCDC) signed contracts for all Pfizer vaccines available for early supply: the government received 3 million doses of COVID-19 Pfizer vaccine 2. April 24, 2021, signing of an additional contract for 40 million doses of Pfizer COVID-19 vaccine for a total of 66 million doses; 3. May 13, 2021, Launch of 835,000 doses of COVAX-AstraZeneca vaccine on May 13, 2021; 4. May 27, 2021, 1.069 million additional vaccine supply contracted by AstraZeneca completing the first half of the year with 10.811/18.38 million doses; 5. June 2021,1 million doses of Janssen vaccine donated in the United States; 6. July 6, 2021, advance supply of 700,000 doses of Pfizer vaccine through a vaccine exchange with Israel; 7. September 1, 2021, introducing 1.5 million doses of vaccine through a health partnership with Romania; 8. September 17, 2021, introducing 871,000 doses of individually contracted Moderna vaccine program, for a cumulative total of 66.38 million doses; 9. October 26, 2021,first introduction of approximately 2,435,000 doses of Moderna vaccine in Korea; 10. November 5, 2021, signing of a contract for 30 million additional doses of Pfizer vaccine for 2022; 11. December 23, 2021, signing of the 2022 Moderna procurement contract for 20 million doses and completion of the mRNA procurement contract for 80 million doses; 12. February 10,2022, Novavax vaccine introduction of 55.1 million; 13. February 11, 2022, introducing 294,000 doses being introduced for a total of 6.35 million doses; 14. April 21, 2022, introducing 492,000 doses of Janssen vaccine; 15. September 27, 2022, introducing a total of 5 million doses of bivalent vaccine based on Moderna BA.1; 16. November 3, 2022, introducing approximately 1.18 million doses of Pfizer BA.4/5 bivalent vaccine in South Korea; 17. January 12, 2023, introducing approximately 400,000 doses of Pfizer infant (6 months to 4 years) vaccine in South Korea
Vaccine development	1. From June 2021, the Public Vaccine Development Center of the National Institute of Infectious Diseases completed biosafety level III certification to fully support the development of the domestic COVID-19 vaccine; 2. June 29, 2022, the first Korean COVID-19 vaccine developed by SK Bioscience, licensed for the SkyCorvione vaccine program by the Ministry of Food and Drug Safety; 3. August 26, 2022, Approved shipment of SkyCoB1 multi-injection vaccine for the vaccination implementation program
Vaccination of minors	1. August 30, 2021 The Expert Committee on Vaccination recommends the inclusion of children and adolescents aged 12–17 years in the vaccination population; 2. March 31, 2022 initial vaccination of children (5–11 years old); 3. February 13, 2023 start of infant Pfizer vaccination of infants (6 months to 4 years) in 1000 individually designated and commissioned healthcare facilities with infant and child treatment and emergency response capabilities
Vaccination booster	**First dose booster vaccination:** 1. December 1, 2012, 1 month for the elderly to receive the 3rd dose intensively; 2. from January 6, 2021, for those aged 18–59 years who have reached the interval between vaccinations, the 3rd vaccination is required in January; 3. from March 21, 2022, 3rd vaccination for adolescents (12–17 years old) **Fourth vaccination for specific groups:** 1. from 14 April 2022, the 4th vaccination for people aged 60 years or older; 2. from July 18, 2022, vaccination of persons over 50 years of age in all age groups more than 4 months after the end of the 3rd vaccination, persons over 18 years of age with underlying disease, residents and staff of facilities susceptible to infection **Corona 19 bivalent vaccination booster dose:** 1. October 11, 2022, start of additional vaccination against COVID-19 in winter; 2. October 27, 2022, bivalent vaccination of adults 18 years and older; 3. December 12, 2022, start of Corona 19 bivalent vaccination for adolescents aged 12–17 years
Vaccination Incentives	Designate the month of November 21 to December 18, 2022 as an intensive winter booster vaccination period, offering incentives based on vaccination status and vaccination rates. First, it is planned to offer discounts on temple stays, discounts on cultural experiences such as free visits to old palaces and tombs, and discounts when using facilities under the jurisdiction of each local government. In addition, incentives will be given to infection-prone facilities and local governments with high infection rates, and points will be added to various assessments and subsidies will be provided.
Compulsory vaccination	From December 16, 2021, the third vaccination is mandatory: 3 months (90 days) after the 2nd vaccination, and the 3rd vaccination must be given within 12 months.
Supporting Policies	1. February 8, 2021, commencement of the registration management function for vaccination of target populations, verification and correction of the list of vaccination recipients at target institutions, and confirmation that target populations can be processed through the system; 2. February 24, 2021,Opening of vaccination management function, where vaccinated people can obtain vaccination certificates on government and vaccination assistant websites after vaccination; introduction of blockchain-based digital vaccination certificates to supplement problems such as forgery and alteration of paper certificates; 3. April 2, 2021, the principle of zero tolerance by social communication teams for key quarantine violations such as not wearing a mask; 4. May 12, 2021, the establishment of "infection control subsidies (fees)" to strengthen quarantine management such as pre-inspection and on-site inspection of multi-purpose facilities to promote quarantine inspection; 5. May 17, 2021, the subsidy for medical expenses for patients with serious illnesses excluded from compensation due to insufficient causality was introduced; 6. June 1, 2021,gradual adjustment of quarantine regulations for vaccinated persons; 7. September 8, 2021,Strengthening of quarantine management, such as the operation of the access control system for medical institutions

The main goal of the Korean government was to achieve universal vaccination as soon as possible in order to control the epidemic and restore normal socio-economic activities. The government has actively promoted the vaccination program by providing free vaccines to the Korean public and ensuring the supply of vaccines to the public through a variety of means, including mass vaccination centers, local vaccination centers, and corporate vaccination. At the public perception level, the Korean public has been supportive of vaccination and widely recognizes the importance of vaccines in controlling the epidemic. The majority of the public actively participated in vaccination and cooperated with the government's vaccination program. At the same time, as Korea focuses on collective consciousness and social responsibility [13], this is also reflected in vaccination, for example, according to the Korean Ministry of Health, the majority of Korean citizens have shown a positive attitude towards the COVID-19 vaccination, and social surveys have shown that a survey of Korean adults showed that more than 80 per cent of respondents expressed their willingness to be vaccinated, and that this positive willingness to be vaccinated reflects Korean citizens' concern for the health of the individual and the community, as well as a sense of responsibility for the control of the outbreak. Korean society has demonstrated a high degree of cooperation and organizational capacity during the COVID-19 vaccination process. The government, medical institutions and community groups have worked together to actively promote the vaccination process and have established an efficient vaccination system, and volunteers have been set up in some communities to help the elderly or people with special needs to make appointments and receive vaccinations. helped ensure the smooth running of the vaccination programme. These reports show the collective consciousness and sense of social responsibility that Korea has demonstrated with regard to the new crown vaccination. Such cultural values have contributed positively to the promotion of a smooth vaccination process. At the same time, in Korean culture, the behaviour of an individual has an impact on the community as a whole, and therefore many people are willing to be vaccinated in order to protect the health of their families and others. Thus, it can be seen that values that emphasize social solidarity and the common good drive public participation in vaccination.Influenced by risk perceptions and values, the majority of the population in Korea had a high risk perception of the severity of COVID-19. Especially during outbreaks, people were more concerned about transmission and health risks and realized that vaccination could reduce the risk of infection and transmission. The government has provided relevant information through scientific media, public education, and expert interpretation to help the public correctly understand the safety and efficacy of vaccines. In general, the Korean government has set the policy goal of universal vaccination and has actively promoted vaccination programs. The public generally recognized the importance of vaccination and was willing to vaccinate in most cases, and cultural factors and values of collective awareness and social responsibility, as well as the perception of risk, also contributed to public participation in vaccination, which together contributed to the vaccination efforts in Korea.

#### Japan

While most countries around the world started vaccinating their populations in early 2021, the Japanese government's delay in doing so has been met with accusations from both inside and outside Japan. Starting with the first mass vaccination on February 17, 2021, the vaccination was carried out in the order of medical-related personnel, the elderly, and the general public, as can be seen by Table 3. Initially limited by not purchasing enough vaccines, vaccination was slow; since the May 2021 contract for Pfizer vaccines, Moderna and AstraZeneca Vaccines, vaccination has accelerated. As of June 20, 2021, 17.8% had completed their first dose and 7.3% had received two complete doses of vaccine, but still well below countries with comparable levels of development. And by June 25 the suspension of workplace vaccinations, which began less than a month ago, was suspended due to a single day's distribution of vaccines reaching its ceiling. Japan's COVID-19 vaccination started late and progressed slowly, showing a stagnant state. One of the major reasons is that Japan has not developed its own vaccine, and the lagging in research and development in Japan, which has always given the image of a "pharmaceutical power", was mainly attributed to the hesitation of Japanese pharmaceutical companies to invest in vaccines and the lack of sufficient budget from the Japanese government to support vaccine development. The second half of 2021 showed another jump in growth rate, which was matched by several vaccine accidents, resulting in a low willingness of the Japanese population to receive vaccinations. Uncertainty about the supply of vaccines and a shortage of syringes in local vaccinations in Japan have slowed progress on vaccinations by most of the time than expected by the Japanese government, which had planned to complete universal vaccination by the end of July before the Tokyo Olympics, but is far behind schedule. In terms of COVID-19 vaccine supply, Japan can only rely on European and American manufacturers. As of June 2021, there were three vaccines approved by the relevant Japanese agencies and allowed to be administered in Japan, namely Pfizer vaccine from the United States, Moderna vaccine from the United States and AstraZeneca vaccine from the United Kingdom. These three vaccines were also on the WHO's "emergency authorization list" and were purchased by many countries, so Japan almost faced the embarrassment of being "out of vaccines" in February–March 2021,when demand in Europe was at its peak. At the same time, vaccination in Japan was free of charge, and once the side effects occurred, the Japanese government paid for it for life, which is one of the reasons why the Japanese government's attitude toward vaccines is very cautious.

**Table 3:** COVID-19 vaccine policies of Japan

Aspects	COVID-19 vaccine policies
Basic vaccination plan	**1. Phase I vaccination:** February 17, 2021 for approximately 40,000 healthcare workers participating in the safety trial; **2. Phase II vaccination:** In mid-March 2021, priority will be given to general medical practitioners, and doctors, nurses, first responders, and health center staff who are responsible for the treatment and handling of COVID-19 will be vaccinated; **3. Phase III vaccination:** Late March 2021 for people aged 65 and older; **4. Phase IV vaccination:** After April 2021, priority will be given to patients with other underlying diseases (people aged 65 with underlying health conditions; people aged 20 to 64 with underlying health conditions with chronic diseases and nursing home staff, etc.); **5. Phase V vaccination:** June 2, 2021 for the general public over 16 years of age (except for specific groups such as pregnant women); **6. Phase VI vaccination:** Other people outside the above phases
Vaccine procurement and supply	1. July 31, 2020, on a basic agreement with Pfizer Inc. for the supply of COVID—19 vaccine; 2. August 7, 2020, on reaching a basic agreement with AstraZeneca Corporation for the supply of COVID—19 vaccines; 3. September 15, 2020, on participation in the global common procurement framework for COVID—19 infectious disease vaccines; 4. October 29, 2020, on the conclusion of vaccine supply agreements with MODERNA and Takeda Pharmaceutical Industries; 5. December 11, 2020, signing of a supply agreement with AstraZeneca for COVID—19 vaccines; 6. January 20, 2021, contract with Pfizer Japan for the supply of COVID—19 vaccine; 7. May 14, 2021,best supply contract with Pfizer Japan for COVID—19 vaccine; 8. June 15, 2021, domestic supply of COVID—19 vaccine to the Socialist Republic of Vietnam; 9. July 20, 2021, agreeement with Takeda Pharmaceutical Industries, Ltd. and MODERNA for the supply of COVID—19 vaccine for next year; 10. September 7, 2021,contract with Takeda Pharmaceutical Co. for the supply ofCOVID—19 vaccine for next year; 11. December 24, 2021, agreement with Takeda Pharmaceutical Industries, Ltd. and MODERNA for the supply of next year's COVID—19 vaccine; 12. February 14, 2022, agreement with Pfizer Japan Inc. for the purchase of additional COVID-19 vaccine this year; 13. March 16, 2022, agreement with Pfizer and MODERNA for the purchase of additional COVID-19 vaccine this year
Vaccine development	1. In May 2020, the Japanese government supplemented the budget of approximately US$1.85 billion for the development and production of COVID-19 vaccine; 2. 21 June 2021, the pace of COVID-19 vaccine development in Japan is accelerated and four biopharmaceutical companies have entered clinical trials; 3. In 2023, Japanese pharmaceutical company Daiichi Sankyo establishes a system capable of producing 20 million doses of COVID-19 vaccine by FY2024, the first plant to develop and apply for approval of an "mRNA" vaccine by a Japanese company
Vaccination of minors	1. August 30, 2022, approval of the Pfizer vaccine booster for use in the 5–11 year old population in Japan; 2. October 24, 2022, infants aged 6 months to 4 years who are registered as residents in Japan become eligible for vaccination; 3. February 28, 2023, vaccination of children 5–11 years of age begins in Tokyo, marking the first vaccination of children in this age group
Vaccination booster	**Third vaccination dose:** 1. September 17, 2021, Japan allowed booster shots to be given 8 months after the second vaccination; 2. November 15, 2021, Japan's Ministry of Health, Labour and Welfare amended its booster vaccination policy, deciding that a booster shot can be given 6 months after the second vaccination, indicating that the vaccine given in the booster shot can be different from the first and second shots; 3. December 1, 2021, the booster vaccination will begin to be available for groups aged 18 and older; 4. April 2022, start of booster vaccination for the 12–17 year old group; 5. August 30, 2022, when Japan approves Pfizer vaccine booster shots for the 5–11 year old population **Fourth vaccination dose:** 1. May 25, 2022, start of the fourth round of vaccination: for people aged 60 years or older and for specific groups aged 18 years or older with underlying disease or a higher risk of severe disease, who also received their first booster dose at least 5 months ago; 2. July 15, 2022, the Japanese government officially expands the fourth vaccination to all medical personnel and staff of facilities for the elderly, etc.; 3. October 20, 2022, the Japanese Ministry of Health, Labour and Welfare decided to shorten the interval between the booster dose of the vaccine against the Omicron variant and the previous dose from 5 to 3 months **Fifth dose of vaccination for specific populations:** October 21, 2022, the fifth vaccination will be administered in Tokyo, Japan, mainly to the elderly and people at higher risk of severe disease
Vaccination Incentives	None
Compulsory vaccination	None
Supporting Policies	Vaccination vouchers are available in Japan. The COVID-19 vaccine can be administered in Japan to the following people who have received a vaccination voucher: Japanese locals aged 5 years or older, mid- to long-term residents, short-term residents who have difficulty returning to Japan (staying in Japan for more than 3 months), and people who are undergoing deportation procedures (people who have not been arrested and are under investigation, or people who have been temporarily released)

The main objective of the Government of Japan was to achieve universal vaccination as soon as possible in order to control the epidemic and restore normal socio-economic activities. The government was actively promoting the vaccination program by providing free vaccines to the Japanese people and ensuring the supply of vaccines to the public by providing services such as appointments and vaccinations through multiple channels. In terms of public perception, Japan has some disagreements with Korea. Influenced by a long history of self-confidence in the strength of medical resources, science and technology, and health education, Japan is more cautious in accepting new things and viewing external information [14], for example, according to a survey of Japanese respondents, only 47 per cent said they would receive the COVID-19 vaccine, while others said they would consider or refuse it; in early 2021, the Japanese government suspended the use of a COVID-19 vaccine because of thrombosis-related complications in some of the recipients. In addition, the COVID-19 vaccine was introduced late and information was not publicized enough, so the public's confidence in the vaccine was weaker than that of Korea, and some members of the public had a wait-and-see attitude toward vaccination. The Japanese have a culture of "non-interference," "consistency between words and deeds," and "following rules," which was also reflected in the vaccination program. In addition, this traditional cultural value also emphasizes safety and stability, which has led the Japanese government to place a high value on the safety and efficacy of vaccines. In Japan, the majority of the population had a high risk perception of the severity of COVID—19. However, when it comes to vaccination, some members of the public have concerns and wait-and-see attitudes about the safety and efficacy of vaccines due to insufficient information disclosure. In addition, the Japanese government has ensured the quality and safety of vaccines by establishing several inspections and norms, which has increased public confidence and sense of security in vaccination. In general, the Japanese government has also taken active measures to promote the vaccination program. The government has promoted and accelerated the vaccination program by strengthening the degree of official information disclosure, increasing public awareness and confidence in the safety of the vaccine, and strengthening related vaccine management.

#### Singapore

Singapore launched its National Vaccination Programme (NVP) on December 30, 2020, with the first vaccines being the Pfizer-BioTech/Comirnaty vaccine, jointly developed by Pfizer and Biotech, the first country in Asia to receive mass vaccination. As can be seen by Table 4, to protect high-risk groups, Singapore's vaccination efforts focus on high-risk groups such as frontline workers and the elderly in the first phase, and then down the list by age, with priority given to the older. The COVID-19 vaccination in Singapore is free for all residents, except for citizens and permanent residents, and foreign nationals with long term residence permits, work permits and student passes. Singapore ranks the lowest in the world with a strength of 0.05% COVID-19 morbidity and mortality rate, and has one of the highest COVID-19 vaccination rates in the world [15]. Meanwhile, Singapore has been at the forefront of vaccine development in Asia, with the COVID-19 vaccine developed in Singapore entering clinical trials in August 2020; the Pfizer vaccine receiving US FDA approval in August 2020; the first batch of Pfizer vaccine arriving in Singapore on December 21, 2020; the country's first healthcare workers receive the vaccine; in March 2022, the two-dose vaccination rate among Singapore's population exceeds 92% and 71% receive a third dose (the vast majority of vaccinations are mRNA vaccines), both leading the world, with 95% of those over 60 years of age completing two doses. It is the Singapore government's firm stance on vaccination that has laid a solid foundation for Singapore to move from zero to living with the virus, and has taught the world a "vivid" lesson by taking the third path between "zero" and "laying flat". The third way between "zero" and "flat" has taught the world a "vivid" lesson.

**Table 4:** COVID-19 vaccine policies of Singapore

Aspects	COVID-19 vaccine policies
Basic vaccination plan	**1. Phase I vaccination:** vaccination of people aged 16 years and older at high risk of infection and those vulnerable to serious illness after infection starting December 30, 2020; **2. Phase II vaccination:** vaccination of older adults, essential services workers, and those at high risk of infection beginning March 8, 2021; **3. Phase III vaccination:** vaccination of persons aged 45 to 59 years starting in April 2021; **4. Phase IV vaccination:** vaccination of people aged 40 to 44 years starting May 19, 2021; **5. Phase V vaccination:** vaccination of Singapore citizens aged 12 to 39 years starting June 10, 2021; **6. Phase VI vaccination:** vaccination for the severely immunocompromised on 28 July 2021; **7. Phase VII vaccination:** vaccination open to selected short-term pass holders from 18 August 2021; **8. Phase VIII vaccination:** other than the above phases
Vaccine procurement and supply	1. May 2020, the Government began discussions with pharmaceutical companies with promising vaccine candidates, having signed pre-purchase agreements with Moderna, Pfizer-BioNTech and Sinovac, and is discussing collaboration with several other pharmaceutical companies; 2. December 21, 2020, Singapore received its first shipment of vaccine from Pfizer-BioNTech; 3. on May 18, 2021, the HSA approved the Pfizer-BioNTech vaccine for use in people aged 12 to 15 years, and the COVID-19 Vaccination Expert Committee approved the extended use of Pfizer-BIONTECH COVID-19 vaccine in people aged 12 to 15 years and the extended dosing interval for MRNA vaccine; 4. June 16, 2021, 24 private healthcare facilities selected to administer government stockpiles of SINOVAC-CORONAVAC COVID-19 vaccine; 5. December 10,2021, MOH has signed a new supply agreement with Pfizer-BioNTech for COVID-19 vaccine; 6. December22, 2021, the first PFIZER-BIONTECH/COMIRNATY COVID-19 vaccine paediatric doses arrived in Singapore; 7. February 14,2022, NOVAVAX NUVAXOVID COVID-19 vaccine approved for use in the national vaccination programme; 8. May 4, 2022, the first doses of NUVAXOVID COVID-19 vaccine arrived in Singapore
Vaccine development	1. March 2020, Vertex Pharmaceuticals, a Singaporean biopharmaceutical company, initiated a Vero COVID-19 vaccine development program in Singapore; 2. July 2020, the VeroCOVID-19 vaccine was tested in clinical trials in Singapore and the results showed that the vaccine was effective in preventing the COVID-19 virus, and Vertex Pharmaceuticals began an aggressive global rollout of the vaccine; 3. Mar 2021, Phase I and Phase II clinical trials of the COVID-19 vaccine "ARCT-021", developed in collaboration with the Duke-NUS Medical School and the US pharmaceutical company Arcturus Therapeutics, have been completed. The results showed that participants developed antibodies against coronary artery disease and no serious adverse reactions were observed
Vaccination of minors	1. Pediatric vaccinations for children 5–11 years of age starting March 16, 2022; 2. Initial vaccination with Moderna/Spikevax vaccine for children 6 months to 4 years of age starting October 25, 2022; booster doses of Pfizer-BioNTech/Comirnaty vaccine for children 5 to 11 years of age
Vaccination booster	**First booster dose:** 1. An additional dose of vaccine for persons aged 60 years and older, residents of elderly care facilities, and persons who are moderately to severely immunocompromised beginning September 15, 2021; 2. A booster program for persons aged 50 to 59 years was initiated on October 3, 2021 in addition to persons aged 60 years and older; 3. booster vaccination for health care workers and health care and frontline COVID-19 workers who completed the primary series vaccination program approximately six months ago, initiated October 9, 2021 4. booster vaccination for all Singaporeans aged 30 and above, permanent residents and long-term pass holders who completed the second dose six months ago from 1 November 2021; 5. Extension of booster vaccination to all individuals aged 18 to 29 years from 14 December 2021; 6. Expansion of the booster vaccination program to those aged 12 to 17 years in early February 2022; 7. One booster dose recommended for individuals 5 to 11 years of age beginning August 24, 2022;
	**Second booster dose:** 1. A second booster dose for people aged 80 years and older and the physically vulnerable from 24 March 2022 2. Vaccination booster for all 12 years and older starting April 22, 2022; Second booster for those 60–79 years of age starting April 22, 2022
	**Corona 19 bivalent vaccination booster:** 1. Bivalent vaccination for those 50 years and older or those not yet minimally protected from October 17, 2022; 2. Bivalent vaccine will be offered to health care workers on October 21, 2022; 3. Bivalent Pfizer-BIONTECH/COMIRNATY vaccination beginning December 12, 2022: All adults 18 years of age and older receive COVID-19 vaccine in a timely manner; those 12 to 17 years of age should also receive an additional dose of bivalent vaccine approximately five months after the last booster dose
Vaccination Incentives	None
Compulsory vaccination	None
Supporting Policies	**1. Ensuring the safety of migrant workers on December 14, 2020:** pilot program to begin in the first quarter of 2021 to allow migrant workers access to the community once a month, subject to compliance with RRT, wearing a contact tracking token and safe living measures; **2. Enhanced safety management measures and surveillance testing on January 22, 2021:** postponement of the National Student Games scheduled for February will be effective January 26, 2021; individuals to limit their visits to a maximum of two additional households per day and limit the maximum number of visitors per household per day to outdoor social gatherings; disallow singing (including diners) and other live performances at food service establishments and work-related events; encourage and other live performances at food service establishments and work-related events; encouraging everyone to connect with family and friends digitally; and increasing enforcement inspections of food service establishments, shopping malls, and other crowded public places; 3. Close monitoring of the safety of the Pfizer-BioNTech COVID-19 vaccine in the population by the Ministry of Health (MOH) and the Health Sciences Authority (HSA) beginning January 2021, and targeting the Vaccine Injury Financial Assistance Program; 4. August 30, 2021 MOH to pilot home isolation, a model of care for fully vaccinated patients with COVID-19 who are not severely symptomatic 5. September 14, 2021 Addition of community care facilities, adjustment of home quarantine policy, and update of electronic quarantine order (eqo), effective September 14, 2021

The Singapore Government's main objective was to achieve universal vaccination as soon as possible to contain the outbreak, to reduce transmission and to protect public health. The Government was actively pursuing the vaccination programme by providing free vaccines to Singaporeans and ensuring the availability of vaccines and vaccination services through the establishment of multiple vaccination centers and the provision of a booking system, among other things. The public in Singapore has been supportive of vaccination and generally recognizes the importance of vaccines in combating epidemics. The majority of the population was willing to be vaccinated and participated in the government's vaccination program. Vaccine-related information has been made widely available to the public, and decisions based on official guidance and expert advice. One of the cultural characteristics of Singapore is the emphasis on social responsibility and the collective good [16], and this value also influences the public's attitude to vaccination for their own health and the health of others. For example, community-based organizations and volunteers in Singapore play an important role in vaccination efforts by providing support and assistance, including organising vaccination points, providing transport arrangements, and assisting the elderly and disadvantaged, etc., in order to ensure that vaccination is easily accessible to all. The co-operation between the Government and the community as well as the participation of the public are important factors contributing to the smooth running of the vaccination programme. In addition, Singaporean society is known for its efficient operations and strict enforcement of regulations, which motivates the government and residents to strictly follow vaccination programs and related measures. The Singapore public had a high risk perception of the severity of COVID-19 and recognized the vaccine as an important means of reducing the spread of infections and diseases. The Government helped the public understand the safety and efficacy of the vaccine by providing accurate and transparent information on the vaccine, thereby increasing public confidence in vaccination. The Government has also stepped up efforts to monitor and regulate the safety of vaccines to ensure public health benefits. In summary, the Singapore government actively promoted vaccination and the public has a positive attitude towards vaccination. The government used policies and resources to ensure vaccine supply and vaccination services, and helped the public to properly understand and participate in vaccination through extensive publicity and provision of accurate information. At the same time, cultural factors and values also contribute to the public's appreciation of social responsibility and strict enforcement of relevant measures. These combined factors have helped Singapore to achieve the goal of universal vaccination, control outbreaks and protect public health.

### The effectiveness of COVID-19 vaccination in these three countries

#### Korea

**Fig. 2:**
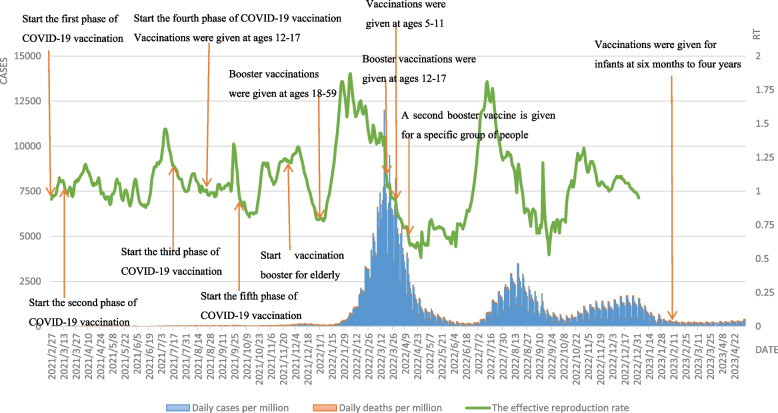
Curves of daily cases per million, daily deaths per million and the effective reproduction rate in Korea.  Note: Daily cases and deaths per million refer to main axis(left). The effective reproduction rate refer to secondary axis(right)

As seen by Fig. 2, the epidemiological curve of COVID-19 in Korea shows that starting from the mass vaccination with COVID-19 at the end of February 2021, Korea experienced a major epidemic peak from February to April 2022 and two minor epidemic peaks from July to September 2022 and from November 2022 to January 2023, respectively. At the peak of the COVID-19 epidemic from February to April 2022, the Omicron variant strain became mainstream, with a sudden increase in daily new cases per million, with new confirmed cases exceeding 170,000 for the first time in a single day on February 12, and a more than tenfold increase in confirmed cases in three weeks, with confirmed cases reaching about 50,000 on consecutive days and more than 230,000 people treated at home. In particular, the number of diagnosed patients over 60 years old increased from 8.0% to 11.7%. Therefore, in order to protect high-risk individuals, the Korean government decided to implement the fourth dose of vaccination for specific groups of people starting from the end of February. However, due to the announcement of the "unsealing" of the epidemic by the Korean government on March 1, 2022, and the large number of students infected on the opening day of the school year on March 2, the cumulative number of infected people in Korea exceeded 10% of the total population by the beginning of March, with the peak of confirmed cases on a single day on March 16, 2022, when the Omicron detection rate reached 100%. Fortunately, the low rate of severe Omicron infections, increased booster vaccination rates in the elderly population and increased use of oral medications slowed the rate of increase in severe cases while the outbreak reached an inflection point, with the number of new confirmed cases quickly falling back to its original state within a month. However, the good times did not last long, and the first confirmed case of BA.2.75, which is more transmissible than BA.5, was found in South Korea on July 15, 2022, but the Korean government did not change the existing epidemic prevention policy, so another small wave was seen in Korea in mid-July 2022. After the winter of November 2022, the epidemic continued to rebound and the "big test" came again. In addition, the Rt value has been fluctuating in a small range around 1 since the mass vaccination in Korea; until the emergence and spread of the Omicron variant in early 2022, the Rt rapidly increased to 1.87 (indicating that one COVID-19 infected person in the above two phases can cause almost two cases of secondary infection), reaching the maximum value since the spread of COVID-19 in Korea. In July 2022, the Rt value increased sharply to the last level due to the arrival of the BA.2.75 mutant strain, but decreased rapidly within one month. Thus, it is easy to see from the development of COVID-19 in Korea that the emergence and spread of new strains still lead to a rapid increase in the number of confirmations and deaths, and that increasing the COVID-19 vaccination rate is an effective means of blocking the spread of the vaccine.

#### Japan

**Fig. 3:**
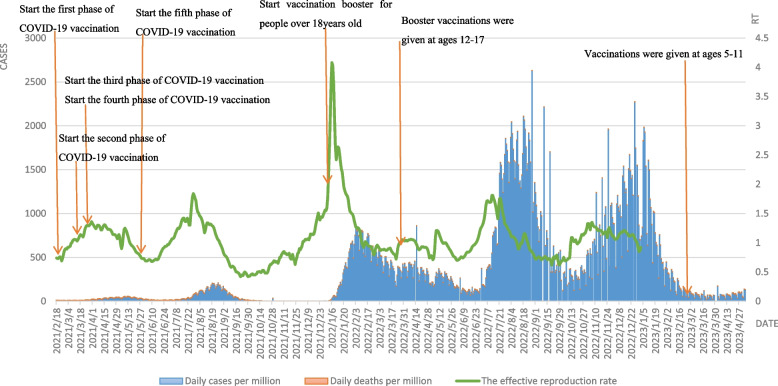
Curves of daily cases per million, daily deaths per million and the effective reproduction rate in Japan. Note: Daily cases and deaths per million refer to main axis(left). The effective reproduction rate refer to secondary axis(right)

In terms of the number of deaths and mortality rates during the wave phase, this round was lower than the previous four waves in Japan, with only 2100 and 0.26%, and the rapid spread of the vaccine played a huge role in this round, as Japan began mass COVID-19 vaccination on February 17, 2021. Except for a small fluctuation in the low level of the epidemic in July and August 2021 due to the Tokyo Olympics, both daily cases per million and daily deaths per million remained at extremely low levels until the end of 2021, and the epidemic in Japan was nearly dissipated after the Delta epidemic. As seen by Fig. 3, in early 2022, with the global spread of the Omicron mutant strain, the sixth wave of the epidemic broke out in Japan, with the first peak of daily cases per million. The Japanese government then announced that adults over 18 years of age who had completed two doses of the basic vaccine could receive a booster, and daily new cases per million steadily declined. The seventh wave of the epidemic broke out in Japan in August 2022. On August 10, 2022,daily new cases per million exceeded 250,000 for the first time, the highest single-day record since the epidemic, and on August 26, 2022,daily new cases per million again broke the record by 260,000, ranking first in the world for two consecutive weeks as the epicenter of the epidemic in children. The Japanese government completely raised the medical system alert level for Omicron to the highest level and approved vaccine booster shots for children aged 5–11 years on August 30, 2022. With the increase in vaccine booster rates, the wave of the outbreak was largely under control by mid to late September 2022. In October 2022, Japan announced the lifting of all control measures, and in November 2022, Japan once again saw the "8th wave of the epidemic", which reached its peak in mid-January 2023 with daily new cases per million similar to the 7th wave. The Rt value in Japan climbed rapidly to 4.08 in a short period of time due to the worldwide epidemic of the Omicron strain at the end of 2021, indicating that one COVID-19-infected individual was able to trigger four cases of secondary infection at that stage. In general, the Rt value fluctuates in a small range above and below the value of 1, and the epidemic is more controllable. It is noteworthy that Japan has achieved good results in this COVID-19 epidemic prevention by occupying 25% of the 378,000 km2 land area with 65-year-olds, while Japan's daily deaths per million are much lower than those in the United States, and steadily remain on the global low level line.

#### Singapore

**Fig. 4:**
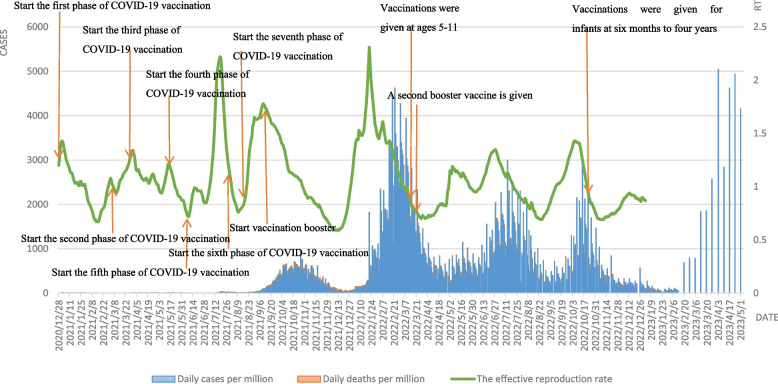
Curves of daily cases per million, daily deaths per million and the effective reproduction rate in Singapore. Note: Daily cases and deaths per million refer to main axis(left). The effective reproduction rate refer to secondary axis(right)

Since the promotion of COVID-19 vaccination, the situation of daily new cases per million varies among countries, with a decrease in daily deaths per million, and the effect is more pronounced in countries with high coverage, but except for Singapore. Vaccination levels in Singapore were very high, with daily new cases per million starting to decline 40 days after vaccination initiation [17]. With 72% of the population fully vaccinated in August 2021, Singapore enters a preparatory period of gradual easing of the vaccination policy, and the vaccination rate reaches 80% in September to enter the second phase of transition, leading to a small peak in daily cases per million around September 2021, with daily deaths per million rising since the end of September and the disease mortality rate increasing from The rate of death increases from 0.1% to 0.3%. In addition, with vaccination already in full swing, Singapore began to shift its thinking on vaccination in June 2021, with the goal of creating a "COVID-resilient nation" and gradually easing social controls, leading to a rapid increase in the Rt value to 2.21 during this period, as seen by Fig. 4. The Rt value increased rapidly to 2.21, and then increased sharply during the implementation of the "differential vaccinator relaxation" in September, 2021, but thanks to Singapore's high vaccination rate, this indicator dropped rapidly in less than a month. On December 19, 2021, the Rt value was the lowest since the spread of COVID-19. The vaccination rate reached 85% in November 2021 and further relaxation of vaccination measures in Singapore, coupled with the sweep of Omicron mutant strains in early 2022, saw a sharp increase in daily new cases per million, reaching the maximum since the COVID-19 epidemic on February 22, and a steady decline in daily new cases per million after the expansion of the vaccination program in early February. In April 2022, the new phase of "living with the virus" was launched, and a series of measures such as exempting international tourists from landing inspection, no longer enforcing the wearing of masks, and eliminating restrictions on the number of people at social gatherings were implemented, leading to two rounds of rebound in a small area of the epidemic and a corresponding increase in Rt values, which gradually decreased as the vaccination rate for the second booster dose increased. Compared with the peak of the previous epidemic, daily new cases per million and daily deaths per million in the second epidemic were almost half of those in the first epidemic, showing that the second booster vaccination had a significant effect on the reduction of daily new cases per million and daily deaths per.

## Discussion

### Comparison and synthesis of vaccination policies and effectiveness in three countries

#### Similarities and differences in vaccination policies in the three countries

Through a comparative analysis of the vaccination policies of the three countries, it can be seen that the three countries share many similarities in their vaccination strategies. Korea, Japan and Singapore all attach great importance to vaccination as an important means of outbreak control, and have developed scientific and rational vaccination strategies and priorities. Based on specific target populations, a phased vaccination program has been formulated, and vaccination priority is determined based on risk assessment to ensure early vaccination of high-risk groups. In terms of vaccine supply and distribution, all three countries were committed to ensuring an adequate supply of vaccines, signing agreements with multiple vaccine manufacturers to ensure the stability of the vaccine supply, thus guaranteeing smooth vaccination, and distributing vaccines through the establishment of vaccination centers and appointment systems. In terms of improving vaccine coverage, Korea, Japan and Singapore have attached great importance to improving vaccination coverage by setting up multiple vaccination centers and providing convenient vaccination services in each region to ensure easy access to vaccinations and vaccinate as many residents as possible.

At the same time, there are differences in vaccination policies. In terms of the challenges faced by the three countries in the early stages of vaccination, Japan faced delays in supply and distribution in the early stages of vaccination, while South Korea and Singapore moved forward with their vaccination programs relatively quickly. In terms of strategies for vaccination, Korea and Singapore adopted mass vaccination centers and appointment systems, while Japan focused more on establishing vaccination sites and strengthening promotion and publicity at the local level. In terms of the speed of vaccination, Korea achieved high nationwide coverage for a large part of the population in a relatively short period of time, while Japan and Singapore reached high coverage at a later stage.

#### Assessment of the effectiveness and impact of different policies in promoting vaccination

A comprehensive analysis of vaccination policies and epidemiological curves shows that in terms of vaccination coverage, Korea, Japan and Singapore have all actively promoted vaccination, and although there are some differences in vaccination speed and strategy, they have all achieved higher vaccination coverage earlier. In terms of outbreak control effects, although all three countries had more than one outbreak peak caused by new mutant strains, with the increase in vaccination coverage, all three countries achieved some outbreak control results, with decreases in daily cases per million, daily deaths per million, and Rt values. Korea's outbreak control after vaccination reached the expected goal and the situation improved significantly; Japan's outbreak control effect gradually appeared, but further efforts are still needed; Singapore successfully controlled the outbreak and reached a more controllable state. In terms of social life and economic recovery, with the advancement of vaccination, people can participate in more social activities, and various industries have begun to recover; in terms of the safety of vaccination, all three countries have implemented stringent vaccine safety testing and regulatory measures, and have rigorously evaluated the quality, safety and efficacy of vaccines, and the vaccines that have been put into use are in line with international standards and regulations, although all three countries Although there are reports of individual adverse reactions and side effects in all three countries, these countries have actively taken measures to detect and manage them, such as setting up reporting systems, strengthening testing and treatment, and other ancillary measures. In terms of ongoing prevention and control measures, the intensity of prevention and control and the degree of implementation varied by country based on the outbreak situation and the level of social compliance, but all three countries emphasized the ongoing implementation of prevention and control measures after vaccination, including non-pharmacological interventions such as social education distances and the use of face masks.

### Analysis of the common logic behind the different policy combinations in the three countries

The ostensibly different vaccination policies of Korea, Japan and Singapore selected for this study show great similarities in vaccination rates and vaccination levels. Therefore, by comparing the anti-vaccination practices of Korea, Japan and Singapore, this study reveals the common logic behind the seemingly very different anti-vaccination approaches and policy combinations of the three countries, i.e., positive willingness to vaccinate, multi-party collaboration, and protection from multiple perspectives are the main threads running through the three countries.

#### Common policy objectives at the core

In terms of vaccine policy objectives, although, due to the differences between countries in terms of epidemic situation, healthcare systems and economic conditions, different countries have chosen different policy instruments and focuses; for example, while accelerating the speed of vaccination, South Korea and Japan have taken measures such as restricting public gatherings in order to control the spread of the cases; while Singapore has adopted means such as retrospection and quarantine. However, in essence, the objective of all three countries is to safeguard public health and safety. First, the general principles of the anti-epidemic institutional arrangements in the three countries are very similar, with epidemic prevention and control planning, national leaders personally directing and taking the lead in vaccination, and all using the risk of infection and serious illness as the first criterion for vaccination consideration; second, in the medical and public health fields, the strength of the resource allocation in Singapore, South Korea, and Japan is at the forefront of the world, and they have played an important role in this epidemic, and during the epidemic, the Singaporean government During the epidemic, the Singaporean government took the initiative to follow up on its social security policies, South Korea had multiple appeals, and Japan vigorously strengthened its medical response system to bridge the infection prevention gap.

#### Public perception and cultural factors are important influences

It is worth mentioning that the public's willingness to vaccinate and vaccine hesitancy have always had an impact on the advancement of COVID-19 vaccination [18]. On the one hand, due to the different vaccination types of countries, their governments have different management and organizational capabilities for vaccination, and the differences in the public's perception of their epidemics and trust in the government may affect the willingness and speed of vaccination in each country. On the other hand, the population's willingness and speed of vaccination are closely related to the risk of the epidemic, Singapore, as a representative of high vaccination rate, has not seen any new local cases for a long time before August 2021 due to extremely excellent epidemic prevention and control ability, the population does not feel the threat of the epidemic and lacks the urgency of vaccination, and by the end of September 2021, even though the number of people fully vaccinated per 100 people has reached 87.00, its case fatality rate began to rise steadily, the epidemic began to circulate since September, and the public began to pay attention to vaccination, which led to a greatly accelerated rate of vaccination, and the epidemic was rapidly brought under control within a month. It can be seen that public perception and cultural factors also have a certain impact on vaccination, Japan under the influence of Confucianism is that many people regard their own health problems as a family and collective responsibility, so they are more cautious in vaccination, and there are more doubts about the effectiveness and safety of the vaccine, which leads to a low rate of vaccination; South Korea's social environment focuses on the spirit of the group, and therefore the South Korean public is Korea's social environment emphasizes group spirit, so the Korean public generally believes that vaccination is a necessary measure and trusts the government's vaccine policy; Singapore's multiculturalism and inclusiveness have led to the public's general belief that vaccination is an action to protect the health of the whole society, and the public is relatively more tolerant and has a higher degree of acceptance of vaccination.

In the practices of Korea, Japan and Singapore, the combination of these factors has contributed to increased vaccination coverage, which in turn has had a significant impact on controlling outbreaks. Therefore, the promotion of COVID-19 vaccination also requires adjusting public perceptions as much as possible based on different cultural factors to reduce the level of public vaccine hesitancy, adequately publicizing the safety and efficacy of vaccination, developing incentives to enhance the motivation of the population to vaccinate, and appropriately conducting mandatory vaccination to increase COVID-19 vaccine coverage.

#### Integrated prevention and control with vaccination and non-pharmacological interventions are indispensable

Non-pharmacological interventions (NPIs) are public health measures aimed at reducing virus transmission by reducing exposure rates, such as contact tracing, international travel controls, closure of public transportation, wearing of masks, closure of workplaces, and cancellation of public events [19]. In this study, Korea, Japan, and Singapore all took many non-pharmacological interventions at the peak of the epidemic before and during the early stages of COVID-19 vaccination, and the designation of policies was combined with governance capacity and the public's willingness and ability to comply with the government's strategies, as well as global epidemic trends, with obvious up- and down- and cross-regional collaboration in Korea and Japan and a weaker ability to collaborate cross-regionally in Singapore due to geographic constraints. But strong grassroots and community-based epidemic prevention capacity helped reduce the impact of the epidemic on the last line of defense. All three countries had strict rule of law and social consensus on severe penalties in the early days, and all demonstrated strong social and technical support by successfully employing surveillance to track close contacts and identify vaccination-appropriate populations.

## Conclusions

This study assessed the effectiveness and impact of different policies in promoting vaccination by examining non-pharmacological interventions during vaccination in three countries and analyzing the vaccination situation in the three countries comparatively, and then explored the impact of factors such as the social, cultural and economic contexts on the implementation and effectiveness of the policies, in order to contribute to a better understanding of the reasons for the differences in the policies between the different regions, as well as the factors behind the same results.

The study found that, despite the different degrees of challenges in vaccine supply and public trust in vaccines in these three countries, they all adopted active vaccination policies, committed to achieving universal vaccination and thus controlling the outbreak, and formulated relevant policies based on the actual situation of the countries in a pragmatic and locally adapted manner, which ultimately led to significant results in controlling the outbreak and protecting public health in all of them. Thus, COVID-19 vaccination is a key public health strategy to reduce the overall burden of COVID-19 globally [20], and the continued promotion of vaccination is a key initiative to control the epidemic, protect public health and restore normal life.

At the same time, we should also recognize that epidemic prevention and control is a multi-task, multi-link complex activity, and there is no need for countries to adopt a one-size-fits-all policy mix, but rather a need to follow a common goal, and it is entirely possible that different combinations of anti-epidemic policies can be formed according to their respective national conditions, which may lead to different paths to the same end. The great difference between East Asia and the rest of the world in this outbreak is that there is some consensus on the basic anti-epidemic goals and logical starting point, as epitomized by Korea, Japan and Singapore. In the global fight against COVID-19, countries can continue to strengthen the implementation of vaccination programmes, drawing on the successful experience of combating the epidemic. At the same time, based on different cultural factors, public perceptions should be adjusted as much as possible so as to reduce the level of public vaccine hesitancy. On this basis, fully promoting the safety and efficacy of vaccination, developing incentives to enhance the public's motivation to vaccinate, and appropriately carrying out mandatory vaccination to enhance COVID-19 vaccine coverage remain the direction for future development.

## Data Availability

The datasets generated and/or analysed during the current study are available in the [Our World in Data] repository, https://ourworldindata.org/.

## Abbreviations

COVID—19: Coronavirus disease 2019.
Rt: The effective reproduction rate
